# On the dispersal of leatherback turtle hatchlings from Mesoamerican nesting beaches

**DOI:** 10.1098/rspb.2011.2348

**Published:** 2012-02-29

**Authors:** George L. Shillinger, Emanuele Di Lorenzo, Hao Luo, Steven J. Bograd, Elliott L. Hazen, Helen Bailey, James R. Spotila

**Affiliations:** 1 Center for Ocean Solutions, Stanford University, 99 Pacific Street, Suite 155A, Monterey, CA 93940, USA; 2 Tag-A-Giant Fund, PO Box 52074, Pacific Grove, CA 93950, USA; 3 School of Earth and Atmospheric Sciences, Georgia Institute of Technology, Atlanta, GA 30332, USA; 4 NOAA Southwest Fisheries Science Center, Environmental Research Division, 1352 Lighthouse Avenue, Pacific Grove, CA 93950, USA; 5 Chesapeake Biological Laboratory, University of Maryland Center for Environmental Science, Solomons, MD 20688, USA; 6 Department of Biology, Drexel University, Philadelphia, PA 19104, USA

**Keywords:** dispersal, eastern Pacific Ocean, leatherback turtle, life history, hatchlings, regional ocean modelling system

## Abstract

So little is known about the early life history of leatherback turtles (*Dermochelys coriacea*) from hatchling to adulthood that this period has been termed the ‘lost years’. For critically endangered eastern Pacific leatherback populations, continued and rapid declines underscore the urgent need to develop conservation strategies across all life stages. We investigate leatherback hatchling dispersal from four Mesoamerican nesting beaches using passive tracer experiments within a regional ocean modelling system. The evolution of tracer distribution from each of the nesting beaches showed the strong influence of eddy transport and coastal currents. Modelled hatchlings from Playa Grande, Costa Rica, were most likely to be entrained and transported offshore by large-scale eddies coincident with the peak leatherback nesting and hatchling emergence period. These eddies potentially serve as ‘hatchling highways’, providing a means of rapid offshore transport away from predation and a productive refuge within which newly hatched turtles can develop. We hypothesize that the most important leatherback nesting beach remaining in the eastern Pacific (Playa Grande) has been evolutionarily selected as an optimal nesting site owing to favourable ocean currents that enhance hatchling survival.

## 1. Introduction

Leatherback turtles (*Dermochelys coriacea*) in the eastern Pacific Ocean have declined by up to 90 per cent over the past two decades and are currently listed as critically endangered [1]. These declines have been driven by a variety of anthropogenic impacts, including development at nesting beaches, poaching of eggs, degradation of foraging habitats and fisheries bycatch [2]. Although conservation efforts at the largest eastern Pacific nesting beach (Playa Grande, Costa Rica) have contributed to turtle protection and recruitment, mortality rates remain high (approx. 22% [2]), owing largely to interactions of juvenile and adult leatherbacks with artisanal and commercial fisheries [3–5]. There is an urgent need to develop integrated management strategies to protect leatherback turtles across all life stages.

The highest-density leatherback nesting colonies remaining in the eastern Pacific occur on beaches within the Parque Nacional Marine Las Baulas (PNMB), Costa Rica (10° 20′ N, 85° 51′ W), although smaller colonies exist throughout the region from Panama to central Mexico [6]. Leatherback turtles at PNMB nest multiple times within a single season (generally December–April) at approximately 8–10 day intervals [7,8]. Most tagging, genetic and mark–recapture studies have focused on the internesting and post-nesting movements of nesting adult females [8–11]. So little is known about the early life history of leatherbacks that the period from hatching to approximately 10 years later, when females return to the nesting beach, is referred to as the ‘lost years’ [12]. Although very little information is available on these early life-history stages, it is well known that hatchlings face a wide range of predators on the beach and within shallow coastal waters [13], and race to get offshore, where decreased predation risk and increased resource availability maximize survival potential [14,15].

The region offshore of the Pacific coast of Mesoamerica is characterized by dynamic ocean conditions. Wintertime winds through coastal mountain gaps contribute to the development of large-scale anticyclonic eddies within the Gulfs of Tehauntepec and Papagayo [16] (electronic supplementary material, figure S1). These are intense and stable features that can last for up to six months and propagate more than 2000 km offshore from the continental margin, transporting nutrient-rich coastal waters and organisms into the ocean interior. In this study, we perform passive tracer experiments within a regional ocean modelling system (ROMS) of the eastern Pacific Ocean to investigate leatherback hatchling dispersal from Mesoamerican nesting beaches. In particular, we test the hypothesis that the leatherback nesting colony at Playa Grande, Costa Rica, is the optimal location in the eastern Pacific for nesting owing to the efficient transport of hatchlings within productive eddies to offshore waters where mortality risks are lower.

## 2. Methods

The modelling framework uses a global eddy-resolving ocean model to provide the boundary conditions for a nested regional ocean model that is used to perform the passive tracer experiments. The global coupled physical–biological ocean model is the Japan Agency for Marine-Earth Science and Technology (JAMSTEC) Ocean General Circulation Model for the Earth Simulator (OFES; see detailed description by Masumoto *et al*. [17]). With a 1/10° horizontal resolution and 54 vertical levels, the OFES model is driven by daily mean forcing from National Centers for Environmental Prediction/National Center for Atmospheric Research (NCEP/NCAR) reanalysis from 1998 to 2010 and with QuikSCAT satellite winds [18]. The ROMS [19,20] is nested within the OFES model within the eastern tropical Pacific (5° S–20° N, 110°–70° W) with the same horizontal resolution and 30 vertical terrain-following layers. The boundary and initial conditions for the ROMS model are provided directly from the OFES simulation. This type of nested ocean modelling approach has been used in previous studies to successfully capture both the mean and the long-term variability of regional-scale Pacific circulations [21–25]. Both the OFES [26] and the ROMS simulations (electronic supplementary material, figure S2) effectively capture the regional dynamics in the eastern tropical Pacific, including high sea surface height variability along the principal eddy pathway, based on comparisons with satellite observations.

The advection and mixing dispersion statistics are diagnosed by injecting a passive tracer at selected coastal regions of the model representing known leatherback turtle nesting grounds [27]: Barra de la Cruz, Mexico (15.8° N, 95.9° W); Playa Chacocente, Nicaragua (11.5° N, 86.2° W); Playa Grande, Costa Rica (10.3° N, 85.9° W); and Playa Carate, Costa Rica (8.4° N, 83.4° W). The dynamics of the passive tracers are implemented in the ROMS model as an advection–diffusion equation with a decay term, following the approach of Combes *et al*. [22]:
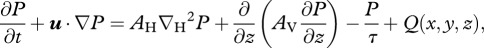


where *P* is the passive tracer concentration, *A*_H_ = 5 m^2^ s^–1^ is the horizontal diffusivity, *A*_V_ the vertical diffusivity obtained by a K profile parametrization scheme [28], *Q* a time-independent source term and *τ* is the decay time scale. The source term is used to release the passive tracer at the selected coastal location by setting *Q* = 1 over a radius of 40 km with a vertical extent of 0–50 m. In this study, we performed release experiments for the years 2000–2008, with tracer continuously released (*Q* = 1) between 15 January and 15 April in each model year, corresponding to the nesting period. In the subsequent months between April and December, we stop the tracer release (*Q* = 0) and evaluate how the tracer concentration is dispersed by the ocean circulation. In order to not accumulate tracer concentration from previous year releases, we set the decay term, *τ*, to a time scale of eight months. Since leatherback hatchlings spend a majority of time in near-surface waters [29], tracer concentrations are integrated through the upper 50 m of the water column. Leatherback hatchlings do not develop full swimming ability for up to six months [29], so the period of the simulation represents their early life history, when they first interact with the ocean environment and can be considered to behave as passive tracers advected by the ocean currents.

## 3. Results

**Figure 1.**
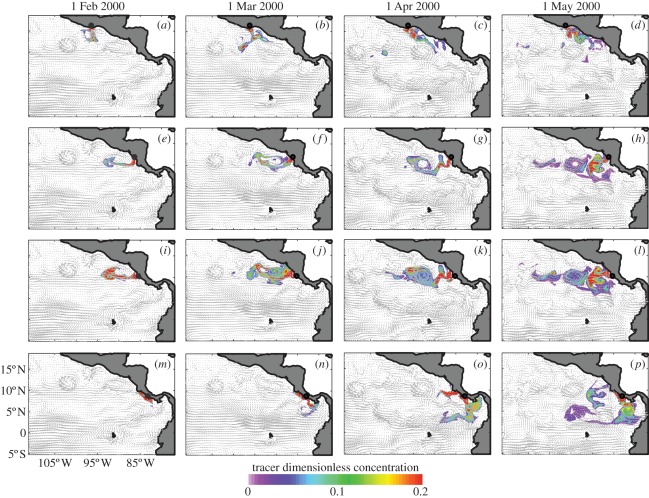
Monthly snapshots of modelled surface circulation (arrows) and tracer concentration (contours) for the year 2000 based on continuous tracer releases between 15 January and 15 April from nesting beaches at (*a*–*d*) Barra de la Cruz, (*e*–*h*) Playa Chacocente, (*i*–*l*) Playa Grande and (*m*–*p*) Playa Carate. Black dots show tracer release locations.

The evolution of tracer distribution following release from each of the nesting beaches shows the impact of eddy transport and coastal currents (figure 1). It is evident that by April 2000, tracer released at Playa Chacocente and Playa Grande is entrained within the eddy pathway and advected offshore, while tracer released from beaches to the north and south is predominantly advected southward and along the coast. Large-scale eddies like those observed in the model output for 2000 were observed in all years for which experiments were performed (2000–2008), consistent with satellite observations [16].

**Figure 2.**
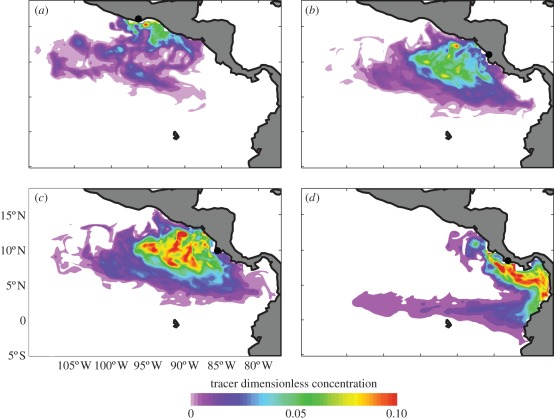
Long-term (2000–2008) mean tracer concentration at 1 June based on continuous tracer releases between 15 January and 15 April from nesting beaches at (*a*) Barra de la Cruz, (*b*) Playa Chacocente, (*c*) Playa Grande and (*d*) Playa Carate. Black dots show tracer release locations.

The long-term mean tracer concentrations confirm that the patterns seen in 2000 were persistent throughout the study period, and that the highest offshore tracer concentrations occurred for tracer released at Playa Grande (figure 2). Significant mean tracer concentration is seen up to 1500 km offshore of Playa Grande by 1 June (approx. 100° W; figure 2*c*), reflecting entrainment within Papagayo eddies throughout the nesting period (cf. figure 1*i–l*). Although offshore tracer dispersal is also seen from Playa Chacocente, again reflecting eddy transport, the highest mean tracer concentrations remain close to the nesting beach (figure 2*b*). In contrast, tracer released at Barra de la Cruz and Playa Carate shows significant nearshore retention (figure 2*a*,*d*), reflecting alongshore transport near the coast throughout the nesting period (cf. figure 1*m*–*p*). Results from the tracer experiments corroborate the hypothesis that hatchlings from Playa Grande are more likely to be entrained and transported offshore by large-scale eddies during the early months following nesting. Although the nesting beaches considered here are separated by only a few hundred kilometres, the evolution of passive tracer distribution, and presumably hatchling turtles, is markedly different, highlighting the intrinsic environmental attributes of Playa Grande as an effective nesting beach.

## 4. Discussion and conclusions

Passive tracer experiments conducted within a ROMS model of the eastern tropical Pacific demonstrate significant offshore eddy transport in late winter from Playa Grande, Costa Rica. This is in contrast to beaches to the north and south, from which there is significant coastal retention. The model results support the hypothesis that hatchling leatherbacks emerging from nests in late winter at Playa Grande can be rapidly and efficiently transported offshore within Papagayo eddies. Because turtles face increased predation risk near the beach, quick offshore transport is likely to increase the probability of survival [14]. Moreover, these eddies provide a productive refuge within which newly hatched turtles can develop.

We hypothesize that Playa Grande was evolutionarily selected as an optimal nesting site owing to enhanced hatchling survival probability as a result of offshore eddy transport. A corollary to this hypothesis is that reduced early-life mortality will lead to increased return of adult females, who show high site fidelity to nesting beaches [30]. Thus, the relatively large leatherback population at Playa Grande compared with other eastern Pacific beaches may be the result of enhanced survival during early life stages. These findings support the suggestion that proximity to favourable ocean currents strongly influences sea turtle nesting distributions [31–33]. Hays *et al.* have also suggested that the prevailing oceanography around nesting sites may be responsible for the selection of foraging sites by adult turtles [4,34].

Understanding the fate of leatherback turtle hatchlings is critical to protect the species. Large marine vertebrates are highly vulnerable to fisheries bycatch because of their late age at maturity and low reproductive rate [4,35]. Because leatherbacks spend more than 10 years at sea before returning to the nesting beach, these ‘lost years’ are potentially critical for population viability. Early attempts to infer the passive drift of animals using ocean models lacked resolution compared with direct measures of passive drift, such as from Lagrangian drifters [36], but these models of ocean circulation are now greatly improved [37]. Models such as those employed here can identify important offshore transport corridors for populations of highly migratory marine species that could be buffered from human impacts. The modelled data can also be coupled with existing tracking datasets involving other leatherback life-history stages (e.g. internesting and post-nesting females, foraging males and subadults) and marine species to examine ecosystem connectivity and to develop integrated life-history management strategies. Directional swimming could have an impact on the resulting trajectory [38], and we therefore plan to incorporate swimming behaviour in the tracer experiments in the future. There are currently only very short-term data available on the swimming behaviour of hatchlings [14,39]. Validation of these models will therefore require new technologies to track leatherback turtles across multiple years and life stages.
